# Offshore Observations of Eastern Red Bats (*Lasiurus borealis*) in the Mid-Atlantic United States Using Multiple Survey Methods

**DOI:** 10.1371/journal.pone.0083803

**Published:** 2013-12-19

**Authors:** Shaylyn K. Hatch, Emily E. Connelly, Timothy J. Divoll, Iain J. Stenhouse, Kathryn A. Williams

**Affiliations:** Biodiversity Research Institute, Gorham, Maine, United States of America; University of Georgia, United States of America

## Abstract

Little is known about the migration and movements of migratory tree-roosting bat species in North America, though anecdotal observations of migrating bats over the Atlantic Ocean have been reported since at least the 1890s. Aerial surveys and boat-based surveys of wildlife off the Atlantic Seaboard detected a possible diurnal migration event of eastern red bats (*Lasiurus borealis*) in September 2012. One bat was sighted approximately 44 km east of Rehoboth Beach, Delaware during a boat-based survey. Eleven additional bats were observed between 16.9 and 41.8 km east of New Jersey, Delaware, and Virginia in high definition video footage collected during digital aerial surveys. Observations were collected incidentally as part of a large baseline study of seabird, marine mammal, and sea turtle distributions and movements in the offshore environment. Digital survey methods also allowed for altitude estimation for several of these bats at >100 m above sea level. These observations provide new evidence of bat movements offshore, and offer insight into their flight heights above sea level and the times of day at which such migrations may occur.

## Background

Nine species of insectivorous bats occur in the northeastern United States, including cave-dwelling bats and migratory tree-roosting bats (hereafter, also “tree bats”) [1]. Tree bats include the eastern red bat (*Lasiurus borealis*), hoary bat (*L. cinereus*), and silver-haired bat (*Lasionycteris noctavigans*). These species tend to be solitary and are known to migrate long distances, rather than hibernating in caves during the winter months [2,3]. However, due to technical limitations associated with tracking the movements of small, highly mobile species over long distances, very little is known of their migratory pathways [4]. Of the three tree bat species, the eastern red bat (hereafter, also “red bat”) is the most frequently encountered species off the Atlantic Seaboard during autumn migration (Table S1). In the winter, this species occurs south of the Ohio River Valley [5] with concentrations along the coastal regions of the Atlantic Seaboard, Bermuda, and the Gulf of Mexico [3,6]. During spring and summer its range expands west to the Rocky Mountains and north into southern Canada [3]. Aside from clues provided by observed mortalities at buildings, wind facilities, collisions with aircraft [3,7-9], and the collection dates and locations of eastern red bat museum specimens, very little is known about the actual migratory movements and pathways of this species [3].

Indirect evidence of the coastal migration of eastern red bats has been acknowledged in the scientific literature for over a century [2,3]. The seasonal appearance of eastern red bats in Bermuda, for instance, indicates their ability to travel long distances (>1000 km) over open ocean [10,11]. Fossil remains of red bats in Bermuda dating back to the middle Pleistocene suggest that this route over the Atlantic Ocean may be an established migratory pathway for this species [12]. Several anecdotal accounts of red bats at sea provide further evidence that this species regularly travels over open ocean and along the outer continental shelf during migration (Table S1). Documentation of calm weather preceding and following bat encounters hundreds of miles offshore suggests that these encounters may be the result of normal migratory movements, rather than weather-driven deviations from land [13,14]. 

Tree bats on migration have recently encountered new threats during this important life stage. While wind energy development is largely viewed as an environmentally friendly energy alternative to fossil fuels, terrestrial wind turbines have been found to cause direct mortality to bats via collisions with the rotors, nacelles, and other infrastructure [7,8,15,16]. Recent post-construction monitoring studies at terrestrial facilities in the U.S. have found that the three northeastern migratory tree bat species constitute 75% of all bat fatalities at terrestrial commercial-scale wind facilities [7], with eastern red bats accounting for the greatest proportion of fatalities in the eastern U.S. [7,8].

Wind energy facilities have been operational in the offshore environment in Europe since 1991 [17]. Despite evidence of bats migrating or foraging up to 80 km offshore in the North and Baltic seas [18,19], there have been no studies to date that have attempted to document collision mortality of bats with offshore wind turbines. However, several studies have stated that such mortality could occur, based on bat behavior offshore [19,20]. Given that eastern red bats are observed more frequently than any other bat species off the Atlantic Seaboard (Table S1), they may be most likely to interact with future offshore wind facilities in this region.

**Figure 1:**
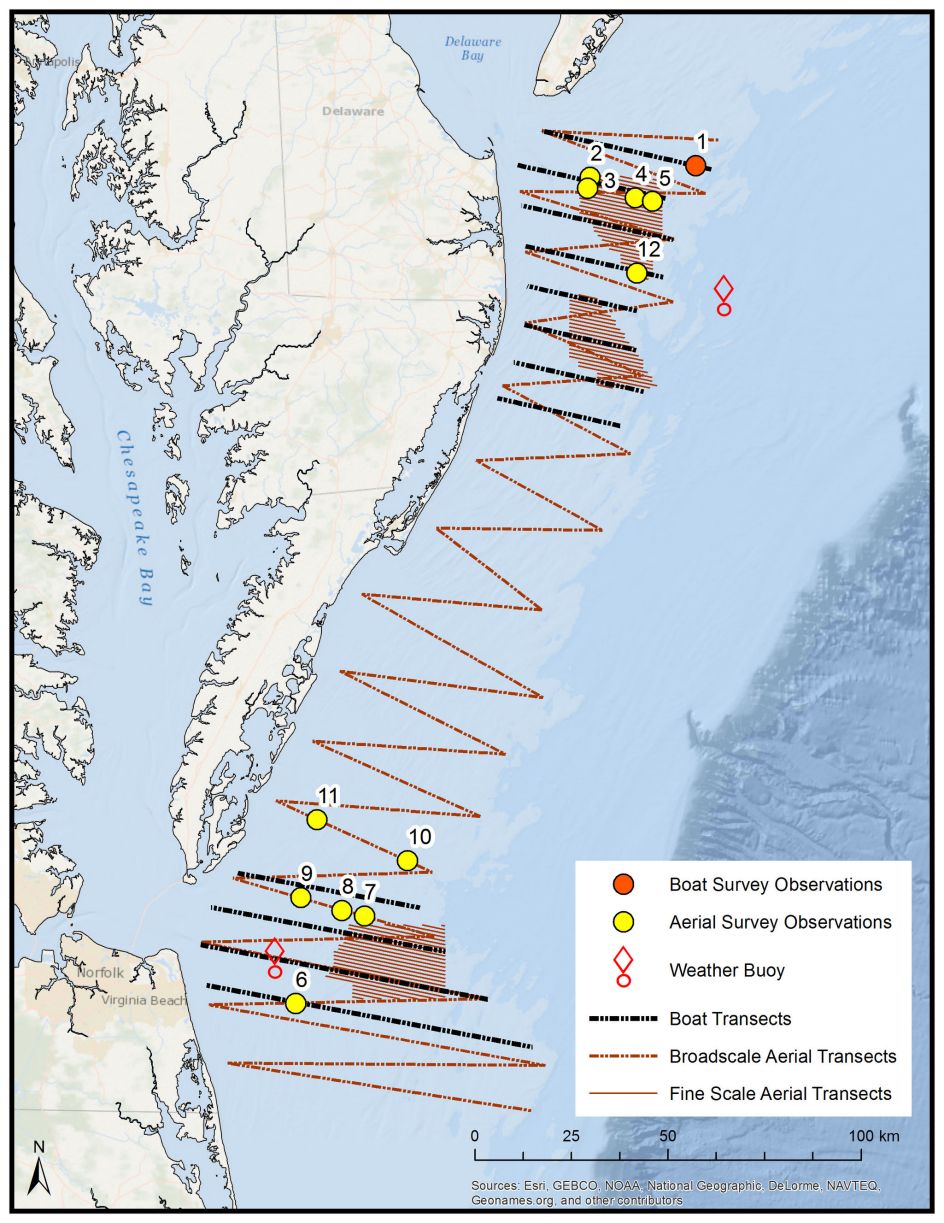
Observations of eastern red bats during aerial and ship-based surveys in the mid-Atlantic U.S (September 2012). Numbers correlate to ID field in Table 1.

Two years of high-definition video aerial surveys and visual shipboard surveys are being conducted as part of a collaborative study of seabird, marine mammal, and sea turtle distributions and movements in a 13,245 km^2^ area off the mid-Atlantic coast of the U.S. (Figure 1). Twelve bats were detected during these surveys in September 2012. These are some of the first reported sightings of bats over the Atlantic Ocean in decades [21]. Here, we present details of these offshore bat sightings, including locations, timing, and estimates of flight height when possible.

## Methods

Surveys were conducted by the Biodiversity Research Institute, the City University of New York, and HiDef Aerial Surveying, Inc., in the first year of a three-year study of wildlife distributions and movements offshore. The study area was focused around three federally designated areas for offshore wind energy development (Wind Energy Areas, or WEAs) off the coasts of Delaware, Maryland, and Virginia (Figure 1).

### Aerial Surveys

Seven aerial surveys were conducted in 2012 including intensive high-density transect coverage within the footprints of the WEAs (20% ground coverage) and a broadscale, lower density coverage of the remainder of the study area (Figure 1). Aerial surveys were conducted at an altitude of 610 m, using an array of four super high-definition (five times HD) video cameras facing forward at 30-45° from vertical in a specially designed air frame secured to the lower fuselage of a twin-engine Cessna aircraft. Cameras captured up to 15 frames per second, and images were duplicated and stored onto a disk array within the aircraft. Cameras with standard telephoto lenses were set to 2 cm ground spatial resolution (GSR). The four cameras had a combined transect strip width of 200 m, and total transect length for each survey was approximately 2,867 km. Survey speed was maintained at approximately 250 km/h and surveys were completed in weather conditions appropriate for observations (<6 Beaufort with no low cloud cover, mist or fog). Recorded images were subsequently reviewed and analyzed by two ground-based teams to locate and mark objects within the footage and to identify animals to lowest taxonomic group. Each object was georeferenced, and bird and bat flight heights were estimated using an extended parallax method. Twenty percent of the data were reanalyzed by auditors, and a successful audit required at least 90% agreement between reviewers and auditors.

**Figure 2:**
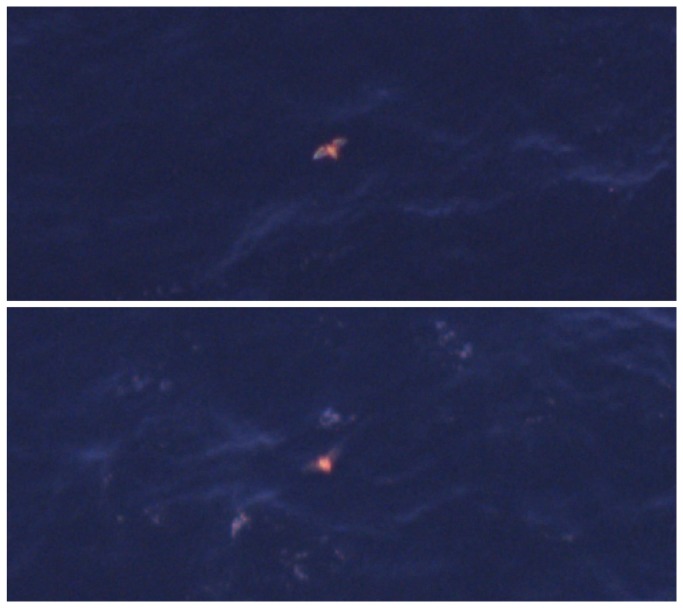
Examples of eastern red bats recorded in high definition aerial footage. Footage recorded from 610 m above sea level with a resolution of 2 cm ground spatial resolution.

Size, color, shape, flight pattern, and clarity of image were used to define species identifications in aerial footage. The eastern red bat was recognizable by its unique coloring falling within the orange-red spectrum. Body shape was characterized by an oblong to oval shape with a generally short and broad appearance, and wings that were more sharply angled proximally to the body when compared to those of a similarly sized bird. Wing color could appear grayish, white, or sometimes clear in video footage due to membranous wing tissue (Figure 2). Confidence levels were assigned to the identification of each animal based on alignment with the above-listed criteria, where “definite” meant >95% certainty, “probable” indicated <95% but >50% certainty, and “possible” indicated <50% certainty in the identification. Identifications with a confidence level of “possible” were excluded from the bat data set presented here. HiDef Aerial Surveying, Ltd. calculated the flying height of bats observed during aerial surveys. HiDef’s flight-height estimation process was based on the measurement of ‘parallax’ in aerial video. Parallax is the apparent motion of an elevated object against a distant background due to the movement of the observer. In the case of an offshore aerial survey, the observer is the camera system, the elevated object is a flying animal, and the background is the surface of the sea. Where the distance to the background and the motion of the observer are known, the parallax is measured from relative positions in digital video frames and used to calculate the height of the object above the background as shown in Equation 1:

$$H=\frac{hd}{l+d}$$

where *H* is the height of the animal, *h* is the height of the aircraft above sea level, *d*is the parallax displacement of the animal measured between two video frames and *l*is the distance travelled by the plane between the two frames (*d*and *l* are both measured in ‘pixels’).

In the specific case of flying animals, the equation above does not hold true because some of the animal’s displacement is due to its own movement rather than that of the observer. There was therefore a need to correct for the animal’s movement before applying the parallax equation to estimate bat flight heights. HiDef used a patented technique for estimating speed and motion of the animal, by analyzing the displacement of the animal (in any direction) compared to the direction of aircraft motion. The component of an animal’s motion in the same direction as the plane was calculated with reference to the animal’s flight direction, which was deduced from the orientation of the animal in the image across multiple frames. This resulted in an estimate of the distance that the animal actually moved between frames, *s* (due to its flight speed), which was then used to correct the observed displacement *d*, enabling the corrected height to be estimated according to Equation 2:

$$H=\frac{h(d-s)}{l+(d-s)}$$

Most objects were observed in at least eight video frames at the altitude and speed at which aerial surveys were conducted. Parallax estimates were then bootstrapped [22] between each pair of frames to produce a confidence interval for the estimated flight height of each animal. Flight height could not be accurately estimated using this approach when the animal was flying parallel to the plane and no displacement was detectable, or the animal was flying at extreme heights and was present in fewer video frames. 

### Ship-based Surveys

Eight boat surveys were conducted by the City University of New York and the Biodiversity Research Institute in 2012. Each survey included twelve transects, spaced 10 km apart, that extended at least one transect north and south of each WEA (Figure 1). Total transect distance was approximately 559 km. During surveys, two teams of experienced observers alternated observation periods and used combined strip and line transect methods to observe and record animals. A continuous watch was maintained by one observer who counted all animals within a 300 m strip to one side of the vessel. The second observer recorded data and observed outside the 300 m strip, primarily to spot cetaceans and sea turtles. Location data were recorded approximately every 30 seconds and each observation was individually georeferenced. For each sighting, the team recorded species, number of animals present, behavior, radial distance from the ship, degree of the animal’s angle to the bow of the ship, direction of movement, and where possible, age and plumage/molt state. Observation conditions were recorded hourly (visibility, sea state). Survey speed was maintained at 18.5 km/h and surveys were completed in sea states appropriate for observations (<5 Beaufort). 

### Weather Data

To examine predictor variables correlated with arrivals and departures of hoary bats at Southeast Farallon Island (SEFI) in a 2007 study by Cryan and Brown [20], we obtained wind speed, wind direction, and barometric pressure data from the closest meteorological buoy (operated by the National Data Buoy Center, or NDBC) for 20:00 Eastern Standard Time (EST) on the night preceding each of our sightings (Figure 1). We determined total moon face illumination for the preceding night using the United States Naval Observatory (USNO) Complete Sun and Moon Data for One Day online database, and checked the National Oceanic and Atmospheric Administration’s (NOAA) Storm Events Database for weather events in states nearest each observation.

### Historic Record Review

We compiled historical records of bats off the Atlantic Seaboard from published literature and museum specimens and included them in Table S1 and Figure S1. Only literature sightings with geographic information were included. For records with descriptive location information, but no geographic coordinates, we estimated positions using Google Earth. Acoustic detections from unknown species were excluded from the data set. We searched fifty museum collections with online access (from the 127 mammal collections included in Cryan 2003[3]) for specimens collected over the Atlantic Ocean. The resulting records were cross-referenced with a list of Atlantic-collected specimens [3] in an effort to incorporate specimens that may not have been accessible online.

### Permits and Approvals

Due to the lack of direct interactions with animals and the altitude at which survey flights were flown, no IACUC approvals were required for this study. During boat surveys, the boat remained on transect at constant survey speed (18.5 km/h), except when complying with National Marine Fisheries Service (NMFS) rules about approaching marine mammals. Due to this survey mode, and to the altitude at which aerial surveys were flown, no permits were required from NMFS to conduct surveys. Surveys complied with all relevant regulations.

## Results

One presumed eastern red bat was observed during a boat-based survey on 6 September 2012, approximately 44 kilometers east of Rehoboth Beach, Delaware, at 09:33 EST (Table 1; 1 in Figure 1). The bat was flying southeast at a distance of approximately 150 m from the ship at a height within normal line of sight. A Beaufort rating of 3 was recorded at the time of the observation. At 20:00 the previous night (5 September 2012), winds at the nearest meteorological buoy (Station 44009) were 8.9 m/sec from the southwest and barometric pressure was 101.04 kilopascals (kPa; Table 1) as opposed to the average autumn wind speeds of 5.77 m/sec from the south southwest and barometric pressure of 101.69 kPa (Table 1). Total moon illumination was 76% and cloud cover data were not available. Tornados, lightning, and thunderstorms occurred in New Jersey and New York on 4 September 2012, but no storm events occurred in Delaware for two days preceding the sighting. 

**Table 1:** Observations of eastern red bats during aerial and ship-based surveys in the mid-Atlantic U.S. in September 2012.

**ID**	**Species**	**Time (EST)**	**Flight Height (m)**	**Fight Height Confidence (%)**	**Travel Direction**	**Land Distance (km)**	**Survey**	**Wind Direction** (°)	**Pressure (kPa)**	**Wind Speed (m/s)**	**Ave. Wind Direction (°) (SD)**	**Ave. Wind Speed(m/s) (SD)**	**Ave. Bar. Pressure (kPa) (SD)**
1	LABO	9:33	-	-	SE	36.6	Boat	205	101.04	8.9	188.24 (97.54)	5.77 (3.28)	101.69 (7.42)
2	LABO	7:56	200 +	100	SW	23.2	Aerial	332	101.72	9.3	188.24 (97.54)	5.77 (3.28)	101.69 (7.42)
3	LABO	8:16	-	-	SW	22.5	Aerial	332	101.72	9.3	188.24 (97.54)	5.77 (3.28)	101.69 (7.42)
4	LABO	8:19	-	-	SW	33.0	Aerial	332	101.72	9.3	188.24 (97.54)	5.77 (3.28)	101.69 (7.42)
5	LABO	8:20	-	-	SW	36.3	Aerial	332	101.72	9.3	188.24 (97.54)	5.77 (3.28)	101.69 (7.42)
6	LABO	8:49	-	-	W	27.2	Aerial	332	101.72	9.3	188.24 (97.54)	5.77 (3.28)	101.69 (7.42)
7	LABO	9:41	200 +	100	SW	38.1	Aerial	341	101.74	10.1	167.4 (99.59)	7.03 (3.8)	101.64 (.66)
8	LABO	9:43	200 +	100	SW	32.3	Aerial	341	101.74	10.1	167.4 (99.59)	7.03 (3.8)	101.64 (.66)
9	LABO	9:45	100 – 200	81	SW	21.8	Aerial	341	101.74	10.1	167.4 (99.59)	7.03 (3.8)	101.64 (.66)
10	LABO	10:13	200 +	100	SW	41.9	Aerial	341	101.74	10.1	167.4 (99.59)	7.03 (3.8)	101.64 (.66)
11	LABO	10:19	200 +	100	SW	16.9	Aerial	341	101.74	10.1	167.4 (99.59)	7.03 (3.8)	101.64 (.66)
12	LABO	10:39	-	-	NW	33.5	Aerial	341	101.74	10.1	167.4 (99.59)	7.03 (3.8)	101.64 (.66)

**ID**s correlate with Figure 1. **Time (EST**) is the time of encounter in Eastern Standard Time. **Travel Direction** indicates bats’ direction of travel. **Land Distance (km**) indicates the distance to nearest landfall in km. **Pressure (kPa**) indicates barometric pressure at sea level; **Wind Speed (m/s**) indicates wind speed averaged over an eight-minute period; and **Wind Direction (°**) indicates direction that wind is coming from in degrees, clockwise from true north during the same period used for wind speed. Wind direction, pressure, and wind speed are presented for 20:00 EST the night prior to the sighting (for comparison with Cryan and Brown 2007) from the National Data Buoy Center (NDBC) buoy with the closest proximity to each bat sighting. **Ave. Wind Direction (°**), **Ave. Wind Speed (m/s**), and **Ave. Bar. Pressure (kPa**) indicate weather data averages and standard deviations (SD) for the NDBC buoys with the closest proximity to each bat sighting from August 1, 2012 - October 31, 2012.

Eleven bats were recorded between 16.9 and 41.8 km off the coasts of New Jersey, Delaware, and Virginia, in footage collected during aerial surveys on 11 September 2012, between 07:56 and 10:39 in the morning (Table 1; Figure 1). Bats #2-6 (Figure 1) were observed between 07:56 and 08:49; at 20:00 on the preceding night, the closest meteorological buoy (Station 44009) measured wind speeds of 9.3 m/sec from the northwest and barometric pressure of 101.72 kPa. Bats #7-12 were observed between 09:41 and 10:39, and at 20:00 on the preceding night meteorological buoy "chlv2" measured wind speeds of 10.1 m/sec from the WNW and a barometric pressure of 101.74 kPa (Table 1). Total moon illumination for 10 September, 2012, was 30% and there were no storm events for the surrounding states of New York, New Jersey, Delaware, Maryland, or Virginia in the two days preceding the sightings. At the time of observation, nine of the 11 bats were moving in a southwesterly direction. A flight height of >200 m was estimated with 100% confidence for five of the 11 bats. Flight height was calculated between 100 and 200 m with 81% confidence for one additional bat. Flight height could not confidently be estimated for the remaining six bats observed during aerial surveys.

In total, twelve presumed eastern red bats were observed offshore in September 2012 in our aerial and boat based surveys. However, no confirmatory specimens were collected and therefore, definitive species identifications were not possible.

For location comparison, 52 accounts of bats offshore of the Atlantic coast of the United States were compiled from published literature and museum specimens, including 11 specimens from museum collections and 41 additional accounts of bats reported in the literature (Table S1).

## Discussion

While bat observations at sea have been reported since the 1890s, ours are among some of the first to be reported in decades (Table S1). The bats observed in our study occurred between 16.9 and 41.9 km from shore and averaged 30 km from the shoreline. Excluding our observations, historic observations of bats occurred between 2.9 and 1949.9 km offshore with a much higher average distance of 103.6 km. Given that these observations were almost all incidental, the relative frequency of occurrence at great distances from land suggests that bats may migrate farther offshore and more often than has been widely acknowledged. 

Several methodologies have been employed for the detection of bats offshore [23]. Previous studies of bat flight altitudes offshore have indicated that they fly close to the water’s surface, even foraging for crustaceans at the water’s surface, and have postulated that bats remain at low altitudes offshore to echolocate off the water and remain oriented [18,19]. However, such studies have been limited in their ability to detect bats migrating at higher altitudes. Griffin’s (1970) and Holland’s (2006) reviews of orientation and navigation in bats indicated that since echolocation is only useful at short ranges, bats likely use vision rather than echolocation to migrate [2,24]. This study is the first that we know of to indicate that bats can fly at much higher altitudes offshore during migration than has been previously supposed. Flying at altitude may allow bats to orient with celestial navigation and follow distinct landscape features, such as the Atlantic Seaboard [24].

Of the six bats observed during aerial surveys for which flight heights were estimable, all six were at altitudes over 100 m above sea level, and five of the six were over 200 m. This may partially reflect the higher likelihood of identification to species for bats flying closer to the cameras. However, the altitude at which high definition aerial surveys were flown, as well as the ability of reviewers to zoom in on objects on their computer screens during identifications, may have allowed for higher detection rates than other survey methods. 

In addition to flying at unexpectedly high altitudes, the bats observed in this study were also migrating at times of day not normally surveyed for bats. The diurnal movements observed in this study may have been due to a lack of landing options offshore. The predominantly southwesterly direction of these bats’ movements when they were observed may support this hypothesis, as they may have been heading towards shore when they were observed. Given the number of bats observed, however, and the clear weather conditions at the time, it is likely that the observed movements were undertaken by choice rather than necessity. Instances of tree bats traveling during autumn migration in “diurnal flocks” have been previously documented [25-27]. Furthermore, while bats likely prefer to migrate at night due to predation pressures [17], there are several anecdotal accounts of bats encountered over the open ocean during the day (Table S1). A recent study found that bats need the sun to calibrate their internal compass and may use this celestial body as a geographic reference [28]. Although high definition aerial surveys are limited to daylight hours, it may be possible to increase detection rates of bat migration events by scheduling surveys during periods of peak migration, when there may be a higher likelihood of detecting these diurnal movements [20] (Table S1). 

The conditions during our observations were largely dissimilar from the predictors of hoary bat arrivals and departures at Southeast Farallon Island (SEFI) in 2007, which were associated with relatively low wind speeds, low moon face illumination, and high cloud cover [20]. Cryan and Brown also found that low barometric pressure was an important predictor for bat arrivals, but not departures [20]. Unlike the migration events on SEFI, our observations corresponded with relatively high northwesterly winds (8.9-10.1 m/s) when compared to the average wind speeds for autumn (Table 1). It is possible the bats observed in the mid-Atlantic were able to fly at such high altitudes by taking advantage of these strong tailwinds, a behavior that has been documented in coastal birds [29]. While moon face illumination was low on the night prior to aerial observations (30%), the boat sighting occurred following a night of high moon face illumination (76%). The bat sighted on the boat survey occurred at a time of relatively low barometric pressure (101.04 kPa), while barometric pressure observed on 11 September was similar to the average pressure for autumn (Table 1). Dissimilarities between predictors of bat arrivals and departures at SEFI and environmental conditions during our study could be due to our small sample size (two days of observations). Our observations took place over the open ocean, while the SEFI bat observations were island-based; this could also account for differences in environmental factors surrounding the observations. For example, low barometric pressure may not be a predictor of movement. Rather, the presence of SEFI may have provided an opportunity for already mobile bats to land if barometric pressure dropped during their flight. 

While bats were not the focus of the 2012 aerial surveys, the high-definition videography survey method proved useful in detecting bats offshore. Results from our surveys are consistent with previous records of *Lasiurus* species thought to be actively migrating, which show a peak occurrence during September [20], and with museum specimens that show a pattern of collection dates indicative of coastal migration in the fall [3]. August boat surveys and October aerial surveys of the same study area in 2012 did not provide further sightings of bats. While groups of bats were not observed, the close proximity and timing of aerial sightings provides further circumstantial evidence of the flocking behavior associated with the autumn migration of *Lasiurus* species [20]. 

Cryan and Brown (2007) suggest that hoary bats, which are normally solitary, convene during autumn migration at the tallest structures on the landscape along their migration routes [20]. If this behavior is also true for the closely related eastern red bat, it is possible that offshore migrants could be attracted to offshore wind facilities where the landscape is otherwise barren of visually distinguishable features. Given the challenges of detecting bat mortalities at offshore wind facilities, more information on bat migration will be required to understand these potential interactions. The expected increase in offshore monitoring of wildlife ahead of U.S. offshore wind power development is likely to provide additional information and observational data on the offshore movements of bats over the next decade.

## Supporting Information

Table S1 **Recent and historic records of bats over the Atlantic seaboard from this study, museum collections, and literature.**
(DOCX)

Figure S1 **Map of recent and historic bat records from Table S1.**
(TIF)
