# Supplementary material for: Offshore Observations of Eastern Red Bats (Lasiurus borealis) in the Mid-Atlantic United States Using Multiple Survey Methods
Source: PLoS One. 2013 Dec 19;8(12):e83803. doi: 10.1371/journal.pone.0083803 (PMC3868561; doi:10.1371/journal.pone.0083803)
Supplement: Table S1 — Recent and historic records of bats over the Atlantic seaboard from this study, museum collections, and literature. (DOCX) [file pone.0083803.s001.docx]

Table S1: Recent and historic records of bats over the Atlantic seaboard from this study, museum collections, and literature.

| **ID** | **Date** | **Species*** | **# bats** | **Sex** | **Time** | **Latitude** | **Longitude** | **Georeference accuracy** | **Distance from shore** | **Method** | **Institution** | **Catalog #** | **Source** |
| --- | --- | --- | --- | --- | --- | --- | --- | --- | --- | --- | --- | --- | --- |
| 1 | 9/6/2012 | LABO | 1 | U | 9:33 | 38.768562 | -74.48744 | actual | 35.4 | sighting | - | - | this study |
| 2 | 9/11/2012 | LABO | 1 | U | 7:56 | 38.741295 | -74.801508 | actual | 22.6 | sighting | - | - | this study |
| 3 | 9/11/2012 | LABO | 1 | U | 8:16 | 38.715514 | -74.809183 | actual | 21.6 | sighting | - | - | this study |
| 4 | 9/11/2012 | LABO | 1 | U | 8:19 | 38.693065 | -74.666917 | actual | 31.0 | sighting | - | - | this study |
| 5 | 9/11/2012 | LABO | 1 | U | 8:20 | 38.684703 | -74.615381 | actual | 35.0 | sighting | - | - | this study |
| 6 | 9/11/2012 | LABO | 1 | U | 8:49 | 36.808896 | -75.648807 | actual | 25.9 | sighting | - | - | this study |
| 7 | 9/11/2012 | LABO | 1 | U | 9:41 | 37.014651 | -75.451316 | actual | 35.6 | sighting | - | - | this study |
| 8 | 9/11/2012 | LABO | 1 | U | 9:43 | 37.027304 | -75.517269 | actual | 30.3 | sighting | - | - | this study |
| 9 | 9/11/2012 | LABO | 1 | U | 9:45 | 37.055766 | -75.637170 | actual | 20.5 | sighting | - | - | this study |
| 10 | 9/11/2012 | LABO | 1 | U | 10:13 | 37.144471 | -75.327618 | actual | 39.6 | sighting | - | - | this study |
| 11 | 9/11/2012 | LABO | 1 | U | 10:19 | 37.237921 | -75.592406 | actual | 15.0 | sighting | - | - | this study |
| 12 | 9/11/2012 | LABO | 1 | U | 10:39 | 38.517294 | -74.663008 | actual | 31.8 | sighting | - | - | this study |
| 13 | "27 November -2 December 2010" | LABO | 3 | U | - | 43.89 | -60.2 | actual | 8.8 | sighting | - | - | [1] |
| 14 | 10/1/2009 | LABO | - | U | 0:04 | 39.73964 | -73.968515 | actual | 10.8 | acoustic | - | - | [2] |
| 15 | 10/1/2009 | MYSP | - | U | 0:06 | 39.73608 | -73.96978 | actual | 10.8 | acoustic | - | - | [2] |
| 16 | 10/1/2009 | LABO | - | U | 0:40 | 39.68844 | -73.989225 | actual | 11.5 | acoustic | - | - | [2] |
| 17 | 9/30/2009 | EPFU/LANO | - | U | 23:17 | 39.627556 | -74.016275 | actual | 12.2 | acoustic | - | - | [2] |
| 18 | 9/30/2009 | MYSP | - | U | 23:59 | 39.746381 | -73.965431 | actual | 10.8 | acoustic | - | - | [2] |
| 19 | 8/31/2009 | LABO | - | U | 3:01 | 39.207816 | -74.381316 | actual | 16.2 | acoustic | - | - | [2] |
| 20 | 8/31/2009 | LABO | - | U | 3:05 | 39.209398 | -74.378081 | actual | 16.2 | acoustic | - | - | [2] |
| 21 | 8/31/2009 | LABO | - | U | 3:29 | 39.22086 | -74.358861 | actual | 15.9 | acoustic | - | - | [2] |
| 22 | 8/31/2009 | MYSP | - | U | 3:33 | 39.22292 | -74.35515 | actual | 15.8 | acoustic | - | - | [2] |
| 23 | 8/31/2009 | EPFU/LANO | - | U | 5:06 | 39.23229 | -74.26682 | actual | 18.6 | acoustic | - | - | [2] |
| 24 | 8/31/2009 | LABO | - | U | 23:27 | 39.47686 | -74.10793 | actual | 13.8 | acoustic | - | - | [2] |
| 25 | 8/31/2009 | LABO | - | U | 23:51 | 39.450551 | -74.128063 | actual | 14.0 | acoustic | - | - | [2] |
| 26 | 8/30/2009 | LABO | - | U | 21:09 | 39.29252 | -74.31913 | actual | 10.7 | acoustic | - | - | [2] |
| 27 | 8/30/2009 | LABO | - | U | 21:19 | 39.291501 | -74.31757 | actual | 10.9 | acoustic | - | - | [2] |
| 28 | 8/30/2009 | LABO | - | U | 22:22 | 39.273665 | -74.284103 | actual | 14.2 | acoustic | - | - | [2] |
| 29 | 8/4/2009 | LABO | - | U | 0:17 | 39.7152083 | -74.062656 | actual | 5.0 | acoustic | - | - | [2] |
| 30 | 8/4/2009 | LACI | - | U | 0:23 | 39.7156416 | -74.0617 | actual | 5.0 | acoustic | - | - | [2] |
| 31 | 8/4/2009 | LABO | - | U | 0:33 | 39.717453 | -74.061705 | actual | 4.9 | acoustic | - | - | [2] |
| 32 | 8/4/2009 | EPFU/LANO | - | U | 0:36 | 39.71497 | -74.0635683 | actual | 4.9 | acoustic | - | - | [2] |
| 33 | 8/4/2009 | EPFU/LANO | - | U | 2:26 | 39.665868 | -74.08599 | actual | 5.2 | acoustic | - | - | [2] |
| 34 | 8/4/2009 | LABO | - | U | 3:19 | 39.669888 | -74.079396 | actual | 5.4 | acoustic | - | - | [2] |
| 35 | 8/3/2009 | LABO | - | U | 0:05 | 39.908871 | -74.02126 | actual | 4.5 | acoustic | - | - | [2] |
| 36 | 8/3/2009 | LABO | - | U | 22:24 | 39.699296 | -74.0832866 | actual | 4.0 | acoustic | - | - | [2] |
| 37 | 8/3/2009 | LABO | - | U | 22:46 | 39.703073 | -74.0786866 | actual | 4.2 | acoustic | - | - | [2] |
| 38 | 8/3/2009 | LABO | - | U | 23:07 | 39.70685 | -74.07438 | actual | 4.0 | acoustic | - | - | [2] |
| 39 | 8/3/2009 | LABO | - | U | 23:45 | 39.712208 | -74.067515 | actual | 4.7 | acoustic | - | - | [2] |
| 40 | 8/3/2009 | LABO | - | U | 23:46 | 39.712241 | -74.06746 | actual | 4.7 | acoustic | - | - | [2] |
| 41 | 8/1/2009 | EPFU/LANO | - | U | 6:13 | 38.8198 | -75.046805 | actual | 4.2 | acoustic | - | - | [2] |
| 42 | 5/2/2009 | EPFU/LANO | - | U | 23:24 | 39.357912 | -74.289357 | actual | 7.9 | acoustic | - | - | [2] |
| 43 | mid October 1969 | LABO | 1 | F | "early morning" | 42.5 | -66.166667 | actual | 99.9 | collection | - | - | [3] |
| 44 | 9/24/1964 | LABO | 1 | U | - | 36.2 | -75.35 | actual | 32.3 | collection | AMNH | 208650 |  |
| 45 | 9/12/1962 | LABO | 1 | F | 12:00 | 42.25 | -67.516667 | actual | 171.2 | collection | MCZ | 50177 |  |
| 46 | 8/25/1953 | LABO | 1 | M | 11:00 | 39.633333 | -70.316667 | actual | 179.2 | collection | - | - | [4] |
| 47 | 8/19/1953 | LANO | 1 | M | 11:00 | 39.6 | -71.05 | actual | 173.6 | collection | MCZ | 58455 | [4] |
| 48 | 10/7/1952 | LABO | 1 | U | - | 42.7 | -62.966667 | actual | 185.9 | collection | - | - | [3] |
| 49 | 9/29/1949 | LABO | ~200 | U | "day break" | 40.166667 | -71 | actual | 117.7 | sighting/ collection | - | - | [5] |
| 50 | 8/25/1938 | LANO | 1 | F | - | 39.15 | -70.366667 | actual | 233.0 | collection | MCZ | 33093 | [6] |
| 51 | 8/25/1938 | LANO | 1 | M | - | 39.15 | -70.366667 | actual | 233.0 | collection | MCZ | 33091 | [6] |
| 52 | 8/25/1938 | LANO | 1 | M | - | 39.15 | -70.366667 | actual | 233.0 | collection | MCZ | 33092 | [6] |
| 53 | 9/7/1937 | UNK | 1 | - | - | 45.116667 | -42.6 | actual | 773.9 | sighting | - | - | [6] |
| 54 | 8/18/1929 | UNK | 1 | - | - | 42.117615 | -70.257639 | estimate | 4.9 | collection | - | - | [6] |
| 55 | 8/17/1929 | LABO | 1 | U | - | 42 | -66 | actual | 154.7 | collection | MCZ | 49662 | [3] |
| 56 | 9/3/1920 | LABO/LANO | 100 | U | - | 35.424689 | -75.136965 | estimate | 29.7 | sighting / collection | - | - | [6] |
| 57 | 9/3/1919 | LABO | 1 | U | 7:07 | 36.970199 | -74.856064 | estimate | 82.3 | sighting | - | - | [7] |
| 58 | 9/7/1918 | LANO | 1 | M | - | 40.687628 | -72.864876 | estimate | 4.5 | collection | AMNH | 182655 |  |
| 59 | 8/20/1913 | LANO | 1 | M | - | 42.552633 | -70.660838 | estimate | 2.9 | collection | MCZ | 14874 |  |
| 60 | 9/6/1907 | LANO | 1 | - | - | 40.474917 | -73.90645 | estimate | 6.6 | sighting | - | - | [6] |
| 61 | "first week of Sept 1902" | UNK | "smaller visit of bat migration" | U | night | 37.552099 | -76.178499 | estimate | 9.9 | sighting | - | - | [8] |
| 62 | "first week of Sept 1902" | UNK | "large migration" | U | night | 39.124307 | -75.225952 | estimate | 9.3 | sighting | - | - | [8] |
| 63 | 9/1/1890 | LABO | 1 | U | - | 40.95046 | -69.432571 | estimate | 54.1 | collection | USNM | 23605 |  |
| 64 | 9/1/1890 | LANO | 1 | U | - | 40.95046 | -69.432571 | estimate | 54.1 | collection | USNM | 23604 |  |

**ID**s correspond to Figure S1. **Date** is the approximate date of data collection. *EPFU=*Eptesicus fuscus,* big brown bat; LABO=*Lasiurus borealis,* eastern red bat; LACI=*Lasiurus cinereus,* hoary bat; LANO=*Lasionycteris noctavigans,* silver-haired bat; MYSP=*Myotis* sp.; UNK=species unknown. **Time** is the approximate time of encounter. **Georeference accuracy** indicates whether latitude and longitude positions in decimal degrees were expressly stated or estimated by the authors using Google Earth. **Method** indicates how data were collected (sighting=visual observation from a boat or other platform; acoustic=passive acoustic detection; collection=specimen preserved in a museum collection). Where applicable, we also list the institution housing the specimen; MCZ= Museum of Comparative Zoology; AMNH= American Museum of Natural History; USNM= National Museum of Natural History, Smithsonian Institution. **Catalog** **#** is the unique identifier assigned to each museum specimen within the institutional collection.

**References**

1. Zenon C, Wong SNP, Willis CKR (2011) Observations of eastern red bats (*Lasiurus borealis*) 160 kilometers from the coast of Nova Scotia. Bat Research News 52: 28–30.
2. Sjollema AL (2011) Bat activity in the vicinity of proposed wind power facilities along the mid-Atlantic coast. Unpublished master’s thesis, Frostburg State University, Frostburg, Maryland.
3. Peterson RL (1970) Another red bat, *Lasiurus borealis*, taken aboard ship off coast of Nova Scotia. The Canadian Field-Naturalist 84: 401.
4. Mackiewicz J, Backus RH (1956) Oceanic records of *Lasionycteris noctivagans* and *Lasiurus borealis*. Journal of Mammalogy 37: 442–443.
5. Carter TD (1950) On the migration of the red bat. Journal of Mammalogy 31: 349–350.
6. Griffin DR (1940) Migrations of New England bats. Bulletin of the Museum of Comparative Zoology 86: 217–246.
7. Nichols JT (1920) Red bat and spotted porpoise off the Carolinas. Journal of Mammalogy 1: 87.
8. Allen GM (1923) The red bat in Bermuda. Journal of Mammalogy 4: 61.
