# Supplementary figures and images for: Offshore Observations of Eastern Red Bats (Lasiurus borealis) in the Mid-Atlantic United States Using Multiple Survey Methods
Source: PLoS One. 2013 Dec 19;8(12):e83803. doi: 10.1371/journal.pone.0083803 (PMC3868561; doi:10.1371/journal.pone.0083803)

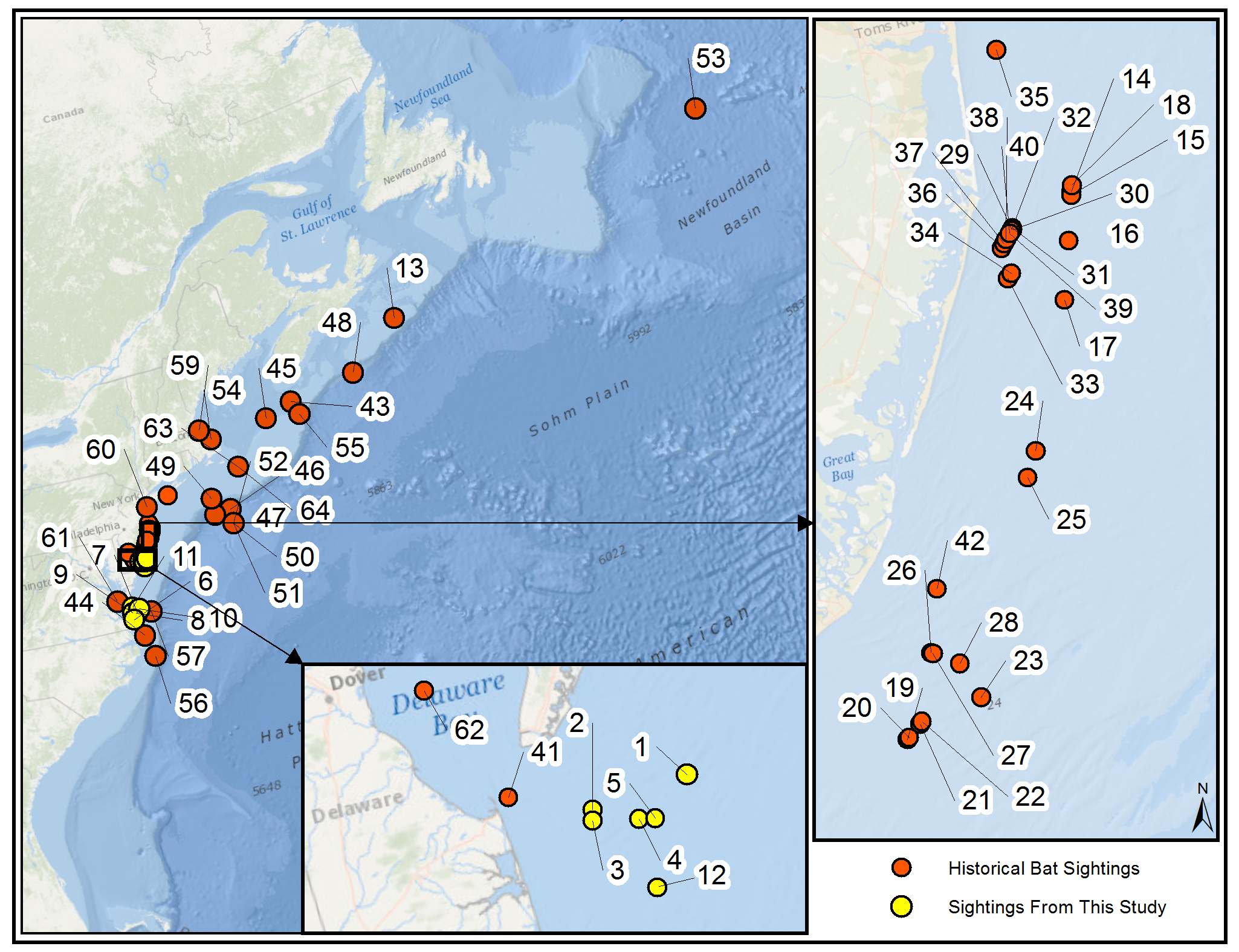

Supplement: Figure S1 — Map of recent and historic bat records from Table S1. (TIF) [file pone.0083803.s002.tif]
